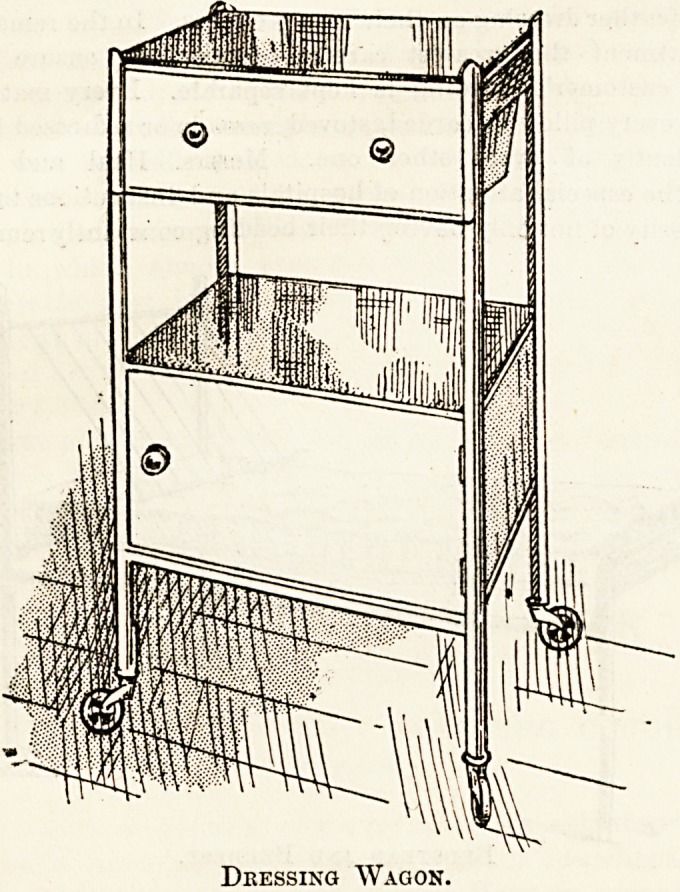# Practical Departments

**Published:** 1905-04-15

**Authors:** 


### PRACTICAL DEPARTMENTS.

### HOSPITAL FURNITURE.

Messrs. Heal and Son, Tottenham Court Road, issue a unique catalogue of furniture for asylums, hospitals, nursing homes, etc. Their bedding, which is practically unrivalled, is made entirely in their own factories, and the manufacturers pride themselves upon being the only retail firm who carry out all the different processes involved in mattress making and feather dressing on their own premises. In the remaking department the greatest care is exercised to ensure that each customer's bedding is kept separate. Every mattress and every pillow is carded, stoved, remade or redressed independently of every other one. Messrs. Heal and Son call the especial attention of hospitals and institutions to the necessity of not only having their bedding constantly remade,

Bedstead and Bedrest.

but of only entrusting the work to firms of high reputation where care is taken to see that it is thoroughly cleansed and purified as well as remade. The firm supplies an enormous assortment of mattresses from brown wool to the best horse-hair, and from lath-spring mattresses to Staple's patent spiral spring mattresses. The latter are strongly recommended as much superior for comfort to any woven wire or chain mattress.

Every kind of bolster and pillow, including the comfortable wedge bolsters, blankets, counterpanes, and quilts, ready-made sheets, and ready-made pillow slips, are to be found on the premises. The bedsteads are legion. A new bedstead for epileptic patients is made of a stout wooden frame, the top edges being stuffed and covered in mackintosh sheeting, and the bottom fitted with studs which drop into slotted plates in the floor. One end is fitted with a lock and key to lock into the floor. The bedstead is provided with a Lawson-Tait spring mattress on a galvanised iron frame. Another epileptic bedstead has a safety sliding rail, with smooth castings and corners. A patent adjustable invalid bedstead has an upper part which may be raised or lowered to any desired angle by turning a handle, without disturbing the occupant. Another bedstead is made throughout of solid-drawn steel tubes and specially-manufactured connections, which, instead of being cast on to the tubes in chills, as in the usual method of manufacture, are bored to fit the tubes accurately, and then either brazed or fastened to the tubes with tapered steel pins. By this method of construction no recesses or angles are formed, as in the case of ordinary bedsteads. A "Labour" bedstead, as made for Queen Charlotte's Hospital, has a bottom in one piece with tubular sides and ends, and three cross-stays to support a removable pine-wood bottom, which is used as a carrying stretcher. Of cots and cribs there are many, including a useful iron folding cot. There are also some "half-side" bedsteads. Messrs. Heal and Son make a special feature of their aseptic furniture. In this department are to be found ward-tables, made of iron, enamelled white, with plate-glass shelves, and rubber-tyred castors; white enamelled iron dressing-wagons, with plate-glass top, and plate-glass cabinet, rubber-tyred castors. There are also enamelled iron lockers, with drawer or drawer and cupboard, plate-glass shelves, and rubber-tyred castors. There is a white enamelled iron chair night-commode, and another in box form. The white enamelled iron cabinets have plate-glass shelves. The firm manufactures a large assortment of bed-rests and bed-tables, also hospital lockers.

Dressing Wagon.

There are some excellent screens of polished deal or bamboo, with removable cretonne curtains. A special feature is made of bedstead accessories, and any bedstead can be made with detachable head or foot-rail, also with a patent self-lifter. The castors and feet of bedsteads are supplied with india-rubber or leather tyres, hardwood or china, also india-rubber ball-feet and india-rubber pad-feet.

## Figures and Tables

**Figure f1:**
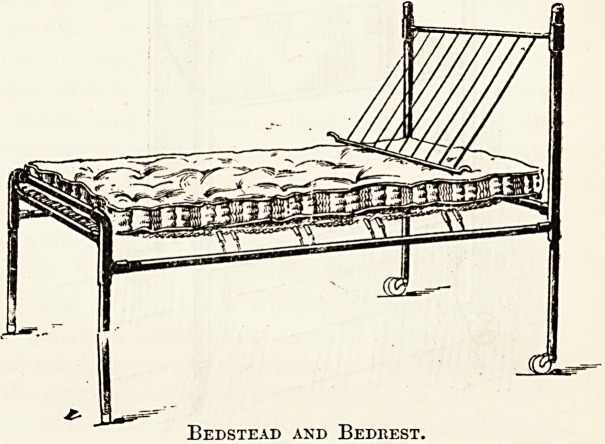


**Figure f2:**